# Dietary patterns and physical activity in the metabolically (un)healthy obese: the Dutch Lifelines cohort study

**DOI:** 10.1186/s12937-018-0319-0

**Published:** 2018-02-12

**Authors:** Sandra N. Slagter, Eva Corpeleijn, Melanie M. van der Klauw, Anna Sijtsma, Linda G. Swart-Busscher, Corine W. M. Perenboom, Jeanne H. M. de Vries, Edith J. M. Feskens, Bruce H. R. Wolffenbuttel, Daan Kromhout, Jana V. van Vliet-Ostaptchouk

**Affiliations:** 1 Department of Endocrinology, University of Groningen, University Medical Center Groningen, HPC AA31, P.O. Box 30001, 9700 RB Groningen, The Netherlands; 2 Department of Epidemiology, University of Groningen, University Medical Center Groningen, PO Box 30001, 9700 RB Groningen, The Netherlands; 3 Lifelines Cohort Study, University of Groningen, University Medical Center Groningen, PO Box 30001, 9700 RB Groningen, The Netherlands; 4 Department of Paramedical Sciences, University of Groningen, University Medical Center Groningen, PO Box 30001, 9700 RB Groningen, The Netherlands; 5 Division of Human Nutrition, Wageningen University, PO Box 17, 6700 AA Wageningen, The Netherlands

**Keywords:** Obesity, Metabolic health, Dietary patterns, Physical activity, Lifestyle

## Abstract

**Background:**

Diversity in the reported prevalence of metabolically healthy obesity (MHO), suggests that modifiable factors may be at play. We evaluated differences in dietary patterns and physical activity between MHO and metabolically unhealthy obesity (MUO).

**Methods:**

Cross-sectional data of 9270 obese individuals (30–69 years) of the Lifelines Cohort Study was used. MHO was defined as obesity and no metabolic syndrome risk factors and no cardiovascular disease history. MUO was defined as obesity and ≥2 metabolic syndrome risk factors. Sex-specific associations of dietary patterns (identified by principal component analysis) and physical activity with MHO were assessed by multivariable logistic regression (reference group: MUO). Analyses were adjusted for multiple covariates.

**Results:**

Among 3442 men and 5828 women, 10.2% and 24.4% had MHO and 56.9% and 35.3% MUO, respectively. We generated four obesity-specific dietary patterns. Two were related to MHO, and in women only. In the highest quartile (Q) of *‘bread, potatoes and sweet snacks’* pattern, odds ratio (OR) (95% CI) for MHO was 0.52 (0.39–0.70). For the healthier pattern *‘fruit, vegetables and fish’*, an OR of 1.36 (1.09–1.71) in Q3 and 1.55 (1.21–1.97) in Q4 was found for MHO. For physical activity, there was a positive association between moderate physical activity and vigorous physical activity in the highest tertile and MHO in women and men, respectively (OR 1.19 (1.01–1.41) and OR 2.02 (1.50–2.71)).

**Conclusion:**

The healthier diet -characterized by *‘fruit, vegetables and fish’*- and moderate physical activity in women, and vigorous physical activity in men may be related to MHO. The (refined) carbohydrate-rich *‘bread, potatoes and sweet snacks’* dietary pattern was found to counteract MHO in women.

**Electronic supplementary material:**

The online version of this article (10.1186/s12937-018-0319-0) contains supplementary material, which is available to authorized users.

## Background

Obese individuals are more likely to develop multiple metabolic complications which increase their risk of type 2 diabetes (T2D) and cardiovascular disease (CVD) [1]. However, some obese individuals, called the metabolically healthy obese, show no sign of conditions associated with the metabolic syndrome (MetS), i.e. impaired glucose metabolism, hypertension and dyslipidaemia [2]. Whether metabolically healthy obesity (MHO) is a truly healthy state remains controversial. Meta-analyses have shown that it is not evident that adults with MHO are at increased risk for all-cause mortality [3], but they still have a risk for T2D and CVD that is intermediate between that of healthy normal weight and unhealthy obese adults [4, 5]. Meaning that even without weight loss, the (cardio)metabolic health can be improved in obese individuals. This is an important finding, given the fact that sustained weight loss is difficult [6, 7].

Interestingly, the MHO phenotype may be modifiable. Several studies with up to 10 years of follow-up data showed that 43.3–47.6% of subjects with MHO transitioned to metabolically unhealthy obesity (MUO) [8–10]. While the ageing process is an important factor, the BioSHaRE-EU Healthy Obesity Project (*n* = 28.077) reported that even the age-standardized prevalence of MHO highly varied between the European countries, 2–19% among men and 7–28% among women, when the same diagnostic criteria for MHO were used [11]. This suggests that on top of age, sex and possibly genes, lifestyle factors are related to the transition from healthy to unhealthy.

The determinants accounting for the metabolic differences observed between MHO and MUO remain uncertain, and particularly data on the role of diet and physical activity are limited. Previous studies on intake of single foods and/or micro- and macronutrients could not find an association with metabolic health subtypes [12–15]. However, as nutrients interact with each other [16], examination of whole diets may be more suitable to gain insight into the relation between diet and metabolic health within the obese population.

In the study of Cahmi et al. [17], adolescents and women with MHO (19–44 years) had higher scores on the HEI-2005 (Healthy Eating Index), which assesses diet quality in relation to U.S. National Dietary Guidelines (2005), compared to the MUO individuals. While the HEI-2005 is a ‘a priori’ dietary pattern based on current knowledge about the role of foods, less is known about the role of existing food consumption patterns within the obese population. Factor analysis is a useful tool to examine these habitual ‘a posteriori’ dietary patterns in a population. Although they do not necessarily represent optimal diets for risk assessment, they are an expression of the way how people eat [15] and are expected to be part of broader lifestyles [16]. Furthermore, they may generate new hypothesis and improve our insight into possibilities for dietary changes for prevention and treatment of metabolic disturbances. A recent systematic review and meta-analysis of observational studies has already shown that ‘a posteriori’ dietary patterns are associated with MetS in the general adult population [18]. In this study, obesity specific dietary patterns will be investigated in relation to metabolic health.

The aim of this study was to evaluate differences in dietary patterns and physical activity between MHO and MUO in the large population-based Lifelines Cohort Study. More specifically, we aimed to 1) generate obesity-specific dietary patterns and examine their associations with demographic- and other lifestyle factors; and 2) compare the dietary patterns and physical activity between MHO and MUO, taking into account among others demographic characteristics, smoking and alcohol use.

## Methods

### Subjects

For this study we used a subset of the cross-sectional Lifelines data, collected between 2006 and 2013. Subjects included in the present study had obesity (body mass index (BMI) ≥30 kg/m^2^), were of western European origin, and aged 30–69 years (*N* = 10,771). In short, Lifelines is a prospective population-based cohort study using a unique three-generation design to study the health and health-related behaviours of 167,729 persons living in the North of The Netherlands. The Lifelines adult population is broadly representative for the adults living in this region [19]. Detailed information on the cohort profile can be found elsewhere [20].

### Clinical measures and definitions

#### Clinical measurements and laboratory methods

Detailed information about the physical examination and biochemical measurements has been published previously [21]. In short, during the first visit measurements of weight, waist circumference, and height (to the nearest 0.5 cm) were performed in light clothing and without shoes. Body weight and height were used to calculate BMI (weight (kg)/height (m)^2^). Blood pressure was measured every minute during a period of 10 min with an automated DINAMAP Monitor (GE Healthcare, Freiburg, Germany), the change in blood pressure within that period can be used as a proxy measure of stress. The average of the final three readings confers the traditional measurement after 5–10 min supine rest and was recorded for systolic and diastolic blood pressure. During the second visit, on average two weeks after the first visit, blood samples were drawn after an overnight fast for measurement of plasma glucose (hexokinase method), high density lipoprotein cholesterol (HDL-C) and triglycerides (respectively, colorimetric method and colorimetric UV method, Roche Modular P chemistry analyser, Basel, Switzerland).

#### Definition of the metabolic health phenotypes

MHO was defined according to the criteria established by the BioSHaRE-EU Healthy Obesity Project [11] this means that subjects with obesity had none of the MetS risk factors, except for waist circumference, according to the revised NCEP ATPIII [22] (using the WHO cut-off of ≥6.1 mmol/L for impaired fasting glucose [23]), and had no previous diagnosis of CVD (defined as self-reported myocardial infarction, stroke, or vascular intervention). MUO was defined as obesity with at least two MetS risk factors, while in ‘intermediate’ obesity only one MetS risk factor was present. Detailed information can be found in Additional file 1.

### Dietary assessment

#### Food frequency questionnaire

We used a self-administered food frequency questionnaire (FFQ) to assess the intake of 110 food items during the last 4 weeks. An existing validated Dutch FFQ formed the basis for the FFQ used in the Lifelines study [24, 25]. The basic Lifelines FFQ focused on estimates of energy intake and macronutrients, including alcohol intake, and comprised all major food groups. For 46 main food items, frequency of consumption was indicated as ‘not this month’ or in days per week or month; including the amount (in units or specified portion size) consumed each time. The FFQ also included 37 questions on consumption of sub-items (e.g. 20^+/^30^+^ cheese, 40^+^ cheese, 48^+^ cheese, or cream cheese) for which frequency was specified as never, sometimes, often and (almost) always. After the first visit the FFQ was filled in by the participant at home and handed in, approximately 2 weeks later, at the Lifelines research center during the second visit for fasting venepuncture. To calculate the participants’ intake the Dutch food composition table of 2006 (NEVO) was used [26].

To correct for potential under- or over-reporting on the dietary questionnaire, extreme values were identified by the top and bottom 2.5% of daily energy intake (kcal/day) and excluded from the dataset. In total, only 0.05% of the number of servings/food items data was missing, while frequency of consumption had been filled in. Food items with missing data on frequency (0.5%) could not be interpreted and were not included in the calculations.

#### Dietary patterns in the obese population

Dietary patterns were derived on the basis of principal components analysis (PCA), a type of factor analysis. With PCA, linear combinations of the originally observed variables are formed by grouping together correlated food groups, identifying underlying components, i.e. dietary patterns, within the data. The correlation coefficients defining these linear combinations represent the loading of each food group on the dietary pattern [27].

Individual food items with a similar nutrient profile and culinary use were combined into 58 food groups (Additional file 2). To test the appropriateness of applying PCA on the study sample, the Kaiser-Meyer-Olkin measurement was conducted for testing sampling adequacy and the Bartlett’s Test of Sphericity for testing the homogeneity of variances.

Dietary patterns were derived on the basis of consumption (g/day) of each food group, unadjusted for energy intake. Within PCA, orthogonal rotation (varimax option) was used to obtain uncorrelated patterns with greater interpretability, which may be used simultaneously in regression models without affecting collinearity [28]. A dietary pattern was retained if the component Eigenvalue was >1.0, by identification of an inflection point in the Scree plot, and interpretability of the dietary pattern. The dietary patterns were considered stable if the same major patterns were identified in two random halves of the data set and per sex group, e.g. the food groups with significant contributions to the dietary pattern (factor loading >0.3 or <−0.3) were similar.

A dietary pattern score was created for each derived pattern by multiplying the loadings with the corresponding standardized intake of the food (calculating Z-scores for men and women separately), and summing across the food groups for each pattern. Dietary patterns were named according to the foods with the highest loading on the pattern (loadings >0.3).

### Physical activity

Physical activity was assessed by the validated SQUASH questionnaire (“Short QUestionnaire to ASsess Health-enhancing physical activity”) [29]. Questions included type of activity, frequency, duration and intensity, referring to a normal week in the preceding months. Metabolic equivalent (MET) values were assigned to activities as defined by the Ainsworth’ compendium of Physical activities [30]. Activities with a MET value of 4 to <6.5 were classified as moderate, and ≥6.5 as vigorous intensity. For statistical analyses we used: the total minutes of moderate and vigorous physical activity per week, the moderate physical activity score and the vigorous physical activity score. The physical activity scores were calculated by multiplying duration (minutes per week) with the MET value, taking into account the intensity. Subjects with implausible values were excluded if: 1) separate activity categories exceeded plausible values; 2) more than two activity categories of the questionnaire were missing; and/or 3) ≥18 h/day was spent on all activities together (this also included light physical activity (2 to <4 MET)) [31].

### Demographic and lifestyle variables

Based on the participants’ responses to the self-administered questionnaires, data were assessed on the presence of diabetes mellitus, history of myocardial infarction, stroke or vascular intervention, current medication use, current use of a (self-)prescribed diet (e.g. energy-, fat- or salt restricted diet, prescribed diet for diabetes or high cholesterol, or fiber rich diet), education level and smoking [21]. Missing values on education level (0.6%) and smoking status (0.2%) were imputed, using single point imputation, with age, sex, netto household income and postal code (as a proxy for social economic status) as predictors.

### Statistics

All analyses were conducted using IBM SPSS Statistics version 22 (IBM Corporation, Armonk, NY, USA). All data are presented for men and women separately. Study characteristics were expressed as percentage (%), means with standard deviation (SD), or as median with interquartile range in case of non-normally distributed data. Differences between groups were tested by *t*-test for continuous variables or Kruskal-Wallis test when appropriate, and Chi-Square test for categorical variables (applying the Bonferroni method). Multivariable logistic regression was used to determine the associations between MHO, dietary patterns (divided in quartiles) and physical activity scores (moderate physical activity score and vigorous physical activity score divided in tertiles) while adjusting for age (categorical) and BMI (dichotomous) (model 1), then smoking (non-, former-, current smoker), education level (low, middle, high), use of a (self-) prescribed diet (yes/no) and energy intake (continuous) were added to the model (model 2). Alcohol use was not considered in the multivariate logistic regression model, because it was an input variable in the PCA. To test for trends the dietary pattern score (using the median value of each quartile), physical activity score, age and BMI was used as a continuous variable. Subjects with MUO were used as the reference group. A two-sided *P*-value ≤0.05 was considered to be statistically significant.

## Results

### Prevalence of metabolic health phenotypes

**Fig. 1:**
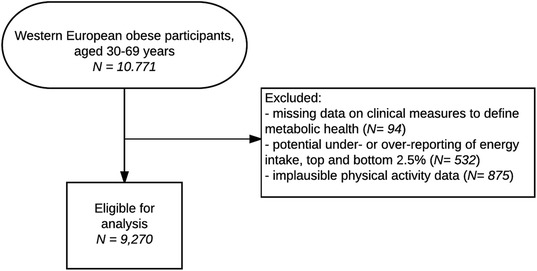
Flow-chart Dietary patterns and PA in MHO

**Fig. 2:**
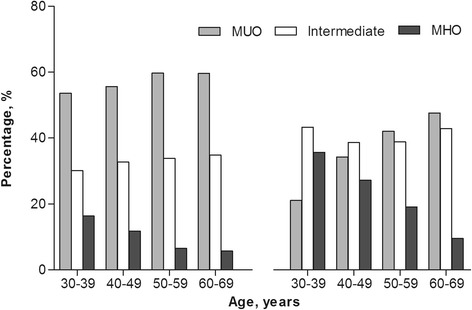
Percentage of the metabolic health phenotype by age groups (left panel men, right panel women)

After exclusion of participants with possible under- or over-reporting of energy intake (*N* = 532), implausible physical activity data (*N* = 875), or missing data on clinical measures (*N* = 94), a total of 9270 obese individuals were included in this study (86.1% from the original sample) (Fig. 1). Of those, 3442 were men (37.1%) and 5828 (62.9%) were women. The prevalence of MHO was 10.2% among men and 24.4% among women (MUO: 56.9% vs. 35.3%, respectively) (Table 1). In general, MHO individuals had a lower BMI and waist circumference than MUO subjects (Table 1). MHO decreased in older age groups for both men and women. Most men were defined as MOU, while most women below the age of 50 years were still defined as ‘intermediate’ obesity (Fig. 2).

**Table 1:** Clinical characteristics according to metabolic health group

	Men			Women		
	MUO	Intermediate	MHO	MUO	Intermediate	MHO
N	1959 (56.9)	1131 (32.9) ^b^	352 (10.2) ^b^	2058 (35.3)	2348 (40.3) ^b^	1422 (24.4) ^b^
Age (years)	49.4 ± 9.4	49.6 ± 9.5	45.3 ± 8.6 ^b^	50.6 ± 9.4	48.3 ± 9.9 ^b^	44.9 ± 8.3 ^b^
BMI (kg/m^2^)	32.2 [30.9–34.3]	31.8 [30.7–33.4] ^b^	31.4 [30.7–32.9] ^b^	33.6 [31.5–36.7]	32.8 [31.2–35.4] ^b^	32.1 [30.9–34.4] ^b^
Waist circumference (cm)	113.0 ± 9.0	111.1 ± 8.2 ^b^	108.9 ± 7.2 ^b^	108.0 ± 10.7	104.0 ± 9.6 ^b^	101.4 ± 8.9 ^b^
Systolic BP (mmHg)	139 ± 14	137 ± 14 ^b^	122 ± 6 ^b^	135 ± 15	131 ± 16 ^b^	118 ± 7 ^b^
Diastolic BP (mmHg)	81 ± 9	81 ± 9	74 ± 5 ^b^	77 ± 9	75 ± 9 ^b^	70 ± 6 ^b^
Fasting glucose (mmol/L)	5.5 [5.1–6.2]	5.2 [5.0–5.6] ^b^	5.2 [4.9–5.5] ^b^	5.5 [5.0–6.3]	5.0 [4.7–5.4] ^b^	4.9 [4.7–5.2] ^b^
HDL cholesterol (mmol/L)	1.02 ± 0.21	1.28 ± 0.25 ^b^	1.33 ± 0.20 ^b^	1.20 ± 0.27	1.48 ± 0.33 ^b^	1.58 ± 0.26 ^b^
Triglycerides (mmol/L)	2.04 [1.60–2.72]	1.21 [0.96–1.50] ^b^	1.08 [0.85–1.34] ^b^	1.76 [1.25–2.21]	1.06 [0.82–1.35] ^b^	0.91 [0.72–1.15] ^b^
Type 2 diabetes * (%)	10.5	0.6 ^b^	0.0	13.3	0.8 ^b^	0.0
CVD history (%)	4.7	4.2	0.0	2.7	1.4	0.0
Use of BP lowering drugs	33.0	20.4 ^b^	0.0	44.4	24.7 ^b^	0.0

Data are presented as mean ± SD or median [interquartile range] or percentage (%). ^a^ denotes a P ≤ 0.01 and ^b^ denotes a P ≤ 0.0001 compared to MUO

*MHO* metabolically healthy obesity, *MUO* metabolically unhealthy obesity, *BMI* body mass index, *BP* blood pressure, *HDL* high-density lipoprotein, *CVD* cardiovascular disease. * Based on known type 2 diabetes and newly-diagnosed type 2 diabetes (defined as a single fasting plasma glucose level ≥ 7.0 mmol/L)

### Dietary patterns

To validate appropriateness of applying PCA on our study sample**,** we calculated the Kaiser Meyer Olkin and Barlett’s Test of Sphericity values. The observed KMO was 0.73 (should not be lower than 0.5) and the BTS was significant (*P* < 0.0001), indicating homogeneity of variance of the foods consumed. Additional file 3 shows the Scree plot of Eigenvalues for each component, i.e. dietary pattern. The Eigenvalues of the components dropped substantially until the fourth component (Eigenvalues > 1.0). As a result, we retained the 4 components solution explaining 18.7% (5.7%, 4.5%, 4.4% and 4.1%, respectively) of the variations in food intake. PCA conducted on the two random halves of the dataset yielded similar results. The same four major patterns were identified for men and women, although the magnitude of the loadings differed more compared to the derived outcomes in the random halves of the dataset.

**Fig. 3:**
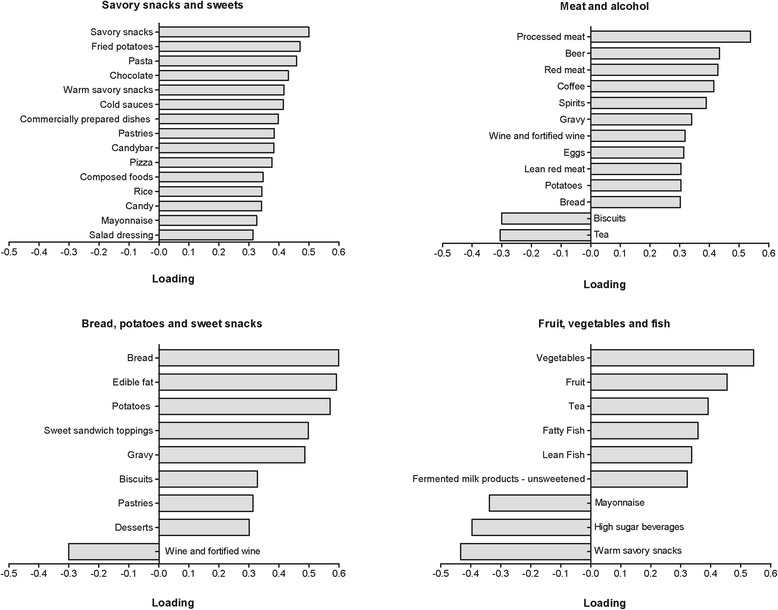
Summary overview of obesity-specific dietary patterns

Positive loadings of the food groups indicate that it is highly correlated with the corresponding dietary pattern, whereas negative loadings are inversely correlated (detailed description Additional file 4). The first dietary pattern we labeled as the *‘savory snacks and sweets’* pattern, the second pattern was labeled as the *‘meat and alcohol’* pattern, the third pattern was labeled the *‘bread, potatoes and sweet snacks’* pattern and the fourth dietary pattern was labeled as *‘fruit, vegetables and fish’* (summary overview Fig. 3). Men with MHO had a higher *‘savory snacks and sweets’* pattern score than men with MUO. No significant differences were found for the other dietary patterns. Compared to women with MUO, women with MHO had a higher score on the *‘savory snacks and sweets’* pattern and the *‘fruit, vegetables and fish’* pattern, but lower scores on the *‘meat and alcohol’* pattern and the *‘bread, potatoes and sweet snacks’* pattern. No differences in total energy intake or the macronutrient intake (% of energy intake) were observed between the three metabolic health phenotypes (Table 2).

**Table 2:** Macronutrient intake, lifestyle factors and education level according to metabolic health phenotype

	Men			Women		
	MUO	Intermediate	MHO	MUO	Intermediate	MHO
N	1959 (56.9)	1131 (32.9) ^b^	352 (10.2) ^b^	2058 (35.3)	2348 (40.3) ^b^	1422 (24.4) ^b^
Energy intake (kcal/day)	2072 ± 488	2088 ± 496	2084 ± 492	1724 ± 443	1722 ± 451	1731 ± 465
Protein (% EI)	15.7 ± 2.3	15.6 ± 2.4	15.7 ± 2.5	16.4 ± 2.7	16.5 ± 2.7	16.4 ± 2.7
Plant	6.1 ± 1.0	6.1 ± 1.0	6.2 ± 1.1	6.1 ± 1.0	6.1 ± 0.9	6.1 ± 1.0
Animal	9.5 ± 2.5	9.4 ± 2.6	9.6 ± 2.8	10.4 ± 2.9	10.4 ± 2.8	10.3 ± 2.9
Carbohydrates (% EI)	45.0 ± 6.1	44.9 ± 6.4	45.2 ± 6.3	46.2 ± 6.3	46.0 ± 6.1	45.9 ± 6.2
Mono- and disaccharides	19.6 ± 5.8	19.5 ± 5.5	19.7 ± 5.3	20.6 ± 5.8	20.6 ± 5.4	20.5 ± 5.2
Polysaccharides	25.4 ± 4.8	25.3 ± 4.8	25.4 ± 4.7	25.5 ± 4.4	25.3 ± 4.5	25.3 ± 4.6
Fat (% EI)	36.0 ± 5.1	36.0 ± 5.1	36.0 ± 4.6	35.9 ± 5.4	35.9 ± 5.2	36.1 ± 5.1
Use of (self-) prescribed diet (%)	5.1	4.9	6.6	12.6	12.2	10.6
Dietary pattern score (Z-scores)						
Savory snacks and sweets	−0.06	−0.01	0.38 ^*^	−0.30	−0.01	0.46 ^b^
Alcohol and meat	0.02	0.04	−0.23	0.08	0.03	−0.16 ^a^
Bread, potatoes and sweet snacks	0.01	0.02	−0.11	0.24	−0.05	−0.25 ^b^
Fruit, vegetables and fish	−0.03	0.05	0.00	−0.13	0.03	0.15 ^a^
MVPA (min/week)	285 [60–960]	360 [120–999] ^a^	360 [140–1337] ^a^	230 [60–600]	270 [100–646] ^a^	242 [90–720] ^a^
Moderate PA score						
T1 ‘low’	45.7	43.4	41.5	48.0	43.2 ^a^	43.1^a^
T2 ‘middle’	21.1	21.9	23.6	20.8	23.2	20.7
T3 ‘high’	33.2	34.7	34.9	31.2	33.6	36.1^a^
Vigorous PA score						
T1 ‘low’	36.1	31.2 ^a^	25.9 ^a^	36.2	33.6	32.5
T2 ‘middle’	33.5	33.7	30.4	30.0	31.0	33.1
T3 ‘high’	30.4	35.1^a^	43.8 ^b^	33.8	35.5	34.4
Smoking (%)						
Non	33.4	40.5 ^a^	47.7 ^b^	41.2	46.8 ^a^	49.2 ^b^
Former	42.0	41.8	36.6	38.6	38.9	37.9
Current	24.6	17.7 ^b^	15.6 ^a^	20.2	14.3 ^b^	12.9 ^b^
Alcohol use (%)						
Non	15.0	9.7 ^b^	9.1^*^	39.2	31.4 ^b^	27.8 ^b^
≤ 2 drinks	70.4	75.5 ^a^	79.3 ^a^	58.0	65.4^b^	70.0 ^b^
> 2 drinks	14.7	14.8	11.6	2.8	3.2	2.3
Education level (%)						
Low	39.7	39.4	36.4	46.8	40.1^b^	32.2 ^b^
Middle	37.8	38.0	41.5	39.3	42.0	43.0
High	22.5	22.5	22.2	13.8	17.9 ^a^	24.8 ^b^

Data is presented as mean ± SD or median [interquartile range]. * denotes a *P* ≤ 0.05, ^a^ denotes a P ≤ 0.01, and ^b^ denotes a *P* ≤ 0.0001. *EI* energy intake, *MUO* metabolically unhealthy obesity, *MHO* metabolically healthy obesity, *MVPA* moderate-vigorous physical active, *PA* physical activity. Education level: low (no formal education, only primary school or intermediate vocational education), middle (higher secondary education) or high (higher vocational education and university)

### Physical activity

Obese men and women with MHO were more moderate-vigorous physically active than subjects with MUO (Table 2). Furthermore, more men with MHO had a vigorous physical activity score in the highest tertile compared to men with MUO (43.8% vs 30.4%, *P* < 0.0001). MHO women had more often a moderate physical activity score in the highest tertile compared to MUO women (36.1 vs 31.2%, *P* < 0.01).

### Smoking, alcohol consumption and education level

Fewer subjects with MHO were current smoker or did not use alcohol compared to subjects with MUO. Men and women with MHO were more frequently light-moderate alcohol consumers (≤2 drinks/day). The distribution of education level was not different between the metabolic health subgroups in men. However, women with MHO were more frequent highly educated (24.8%) compared to women with MUO (13.8%) (*P* < 0.0001) (Table 2).

### Demographic, physical activity and other lifestyle characteristics within dietary patterns

Because dietary patterns are part of a broader pattern of lifestyle factors, demographic and lifestyle characteristics are shown by quartiles of dietary pattern scores (Table 3). We also checked the dietary pattern scores across age groups (Additional file 5). Subjects in the highest quartile of the *‘savory snacks and sweets’* pattern, were of a younger age, more often metabolically healthy obese, higher educated and had less often a chronic condition (T2D, CVD history, or hypertension) or used a (self-) prescribed diet. At the same time, they were less physically active and more often smokers. Scores on both the *‘meat and alcohol’* pattern and the *‘bread, potatoes and sweet snacks’* were stable across age groups, but subjects with a high score were less often metabolically healthy obese and less educated compared to subjects with a low score. The *‘meat and alcohol’* pattern was, furthermore, characterized by less physical activity, smoking and heavy alcohol use (19.0% consuming >2 drinks/day). In contrast, subjects in the highest quartile of the *‘bread, potatoes and sweet snacks’* pattern were more physically active, less often a smoker and drank less alcohol. Older people were more likely to fit the *‘fruit, vegetables and fish’* pattern. The pattern was associated with higher education levels, more physical activity, less smokers (although more former smokers) and less alcohol use. However, chronic conditions and use of a (self-) prescribed diet was more prevalent in the highest tertile.

**Table 3:** Demographic, physical activity and other lifestyle characteristics within the first and fourth quartile of the dietary pattern

	Savory snacks and sweets		Meat and alcohol		Bread, potatoes and sweet snacks		Fruit, vegetables and fish	
	Q1	Q4	Q1	Q4	Q1	Q4	Q1	Q4
N	2317	2317	2317	2317	2317	2317	2317	2317
Males (%)	860 (37.1)	860 (37.1)	860 (37.1)	860 (37.1)	860 (37.1)	860 (37.1)	860 (37.1)	859 (37.1)
Age (years), mean ± sd	53.9 ± 9.9	44.2 ± 7.4 ^b^	47.9 ± 10.1	48.7 ± 8.9	49.0 ± 9.5	48.4 ± 9.8	44.8 ± 8.1	51.6 ± 9.9 ^b^
BMI (kg/m^2^), median [IQR]	33.5 [30.9–34.7]	32.6 [31.1–35.3] ^a^	32.5 [30.9–34.8]	32.5 [31.0–35.2]	32.4 [30.9–34.8]	32.4 [30.9–34.9]	32.7 [31.1–35.4]	32.3 [30.9–34.6] ^b^
Metabolic health (%)								
MUO	46.4	40.1 ^b^	42.1	43.8	39.4	44.9 ^a^	44.8	41.3
Intermediate	38.6	37.5	36.3	37.9	38.5	37.4	36.7	38.9
MHO	15.0	22.4 ^b^	21.7	18.3 ^a^	22.1	17.7 ^a^	18.5	19.8
Energy intake (kcal/day)	1486 ± 412	2279 ± 411^b^	1572 ± 449	2191 ± 456 ^b^	1504 ± 432	2256 ± 404 ^b^	1973 ± 511	1813 ± 490 ^b^
MVPA (min/week)	280 [110–720]	255 [90–769]	268 [90–720]	280 [90–780]	250 [75–660]	300 [100–840] ^b^	270 [60–960]	290 [120–680]
Moderate PA score	225 [0–1350]	300 [0–2625] ^a^	240 [0–2025]	300 [0–1700] ^a^	300 [0–1700]	300 [0–2700] ^a^	300 [0–3780]	300 [0–1500] ^a^
Vigorous PA score	960 [105–2160]	645 [0–1600] ^b^	800 [48–1920]	640 [0–1680] ^b^	720 [0–1800]	780 [80–1920] ^a^	480 [0–1440]	960 [240–2160] ^b^
Education level (%)								
Low	52.1	29.8 ^b^	37.1	41.4 ^a^	32.2	46.9 ^b^	47.5	32.8 ^b^
Middle	33.4	45.7 ^b^	41.1	40.5	40.1	39.4	41.1	38.5
High	14.5	24.5 ^b^	21.8	18.1 ^a^	27.7	13.8 ^b^	11.4	28.6 ^b^
Smoking (%)								
Non	38.6	46.3 ^b^	54.4	31.3 ^b^	38.6	46.6 ^b^	39.0	44.2 ^a^
Former	45.0	34.0 ^b^	34.9	42.8 ^b^	42.4	37.5 ^a^	30.3	46.7 ^b^
Current	16.4	19.7 ^a^	10.7	25.9 ^b^	19.0	15.9 ^a^	30.7	9.1 ^b^
Alcohol use (%)								
Non	30.0	23.4 ^b^	39.3	14.3 ^b^	16.9	32.8 ^b^	29.6	22.4 ^b^
≤ 2 drinks/day	63.9	68.3^*^	60.1	66.6 ^b^	69.5	64.2 ^a^	59.8	71.0 ^b^
> 2 drinks/day	6.1	8.2 ^*^	0.6	19.0 ^b^	13.6	3.0 ^b^	10.6	6.6 ^b^
(Self-) prescribed diet (%)	18.2	3.9 ^b^	11.8	7.0 ^b^	15.8	5.2 ^b^	3.5	18.9 ^b^
Type 2 diabetes (%)	10.3	2.8 ^b^	5.6	5.0	4.5	5.4	3.2	6.6 ^b^
CVD history (%)	4.4	1.2 ^b^	2.2	2.4	2.6	2.8	1.8	3.4 ^a^
Use of BP lowering drug (%)	37.3	17.7 ^b^	24.3	25.2	25.2	24.5	19.5	29.4 ^b^

Data is presented as mean ± SD or median [interquartile range] or percentage (%). ^*^ denotes a P ≤ 0.05, ^a^ denotes a P ≤ 0.01 and ^b^ denotes a P ≤ 0.0001 compared to Q1 of the same dietary pattern. *BMI* body mass index, *MUO* metabolically unhealthy obesity, *MHO* metabolically healthy obesity, *MVPA* moderate–vigorous physical activity, *PA* physical activity, *CVD* cardiovascular disease, *BP* blood pressure. Education level: low (no formal education, only primary school or intermediate vocational education), middle (higher secondary education) or high (higher vocational education and university)

### Dietary patterns and physical activity as determinants of MHO

Of the four obesity-specific dietary patterns, two patterns were associated with MHO and in women only. Among women, a higher score on the *‘fruit, vegetables and fish’* pattern was associated with a higher OR for MHO (P for trend <0.0001) (Table 4, model 1 and 2). In contrast, higher scores on the ‘*bread, potatoes and sweet snacks*’ pattern was associated with a lower OR for the MHO phenotype (P for trend <0.0001). A higher moderate physical activity score and a higher vigorous physical activity score was associated with a higher OR for MHO in women (P for trend 0.020, model 2) and men (P for trend 0.0001, model 1 and 2), respectively.

**Table 4:** Multivariable-adjusted odds ratios for the associations of dietary patterns and physical activity with metabolically healthy obesity

	Men		Women	
	Model 1	Model 2	Model 1	Model 2
Dietary pattern				
Savory snacks and sweets				
Q1	1	1	1	1
Q2	1.12 (0.78–1.63)	1.18 (0.79–1.73)	**1.26** (1.01–1.57)	1.16 (0.92–1.46)
Q3	**1.46** (1.02–2.09)	**1.57** (1.05–2.36)	1.13 (0.90–1.42)	1.00 (0.77–1.29)
Q4	1.29 (0.88–1.90)	1.41 (0.86–2.30)	**1.43** (1.13–1.81) ^a^	1.24 (0.90–1.71)
*P for trend*	NS	NS	0.045	NS
Meat and alcohol				
Q1	1	1	1	1
Q2	0.89 (0.64–1.23)	0.98 (0.70–1.36)	0.98 (0.80–1.21)	1.05 (0.85–1.30)
Q3	0.78 (0.56–1.09)	0.91 (0.64–1.30)	0.83 (0.67–1.03)	0.88 (0.71–1.10)
Q4	0.72 (0.51–1.02)	0.94 (0.63–1.42)	1.02 (0.82–1.26)	1.14 (0.89–1.46)
*P for trend*	0.045	NS	NS	NS
Bread, potatoes and sweet snacks				
Q1	1	1	1	1
Q2	0.78 (0.56–1.09)	0.77 (0.54–1.09)	**0.64** (0.52–0.80)	**0.64** (0.51–0.80) ^b^
Q3	0.75 (0.54–1.06)	0.76 (0.52–1.10)	**0.66** (0.53–0.81)	**0.66** (0.52–0.83) ^a^
Q4	0.92 (0.66–1.28)	0.92 (0.59–1.44)	**0.55** (0.44–0.68)	**0.52** (0.39–0.70) ^b^
*P for trend*	NS	NS	<0.0001	<0.0001
Fruit, vegetables and fish				
Q1	1	1	1	1
Q2	1.03 (0.75–1.44)	1.01 (0.73–1.41)	**1.24** (1.01–1.54)	1.14 (0.91–1.41)
Q3	0.88 (0.62–1.24)	0.83 (0.57–1.18)	**1.55** (1.25–1.92) ^b^	**1.36** (1.09–1.71) ^a^
Q4	0.96 (0.68–1.36)	0.87 (0.60–1.25)	**1.75** (1.40–2.19) ^b^	**1.55** (1.21–1.97) ^a^
*P for trend*	NS	NS	<0.0001	<0.0001
Moderate PA score				
T1 ‘low’	1	1	1	1
T2 ‘middle’	1.22 (0.90–1.65)	1.18 (0.87–1.61)	1.10 (0.90–1.34)	1.08 (0.88–1.31)
T3 ‘high’	1.03 (0.78–1.35)	0.99 (0.75–1.32)	1.15 (0.97–1.36)	**1.19** (1.01–1.41)
*P for trend*	NS	NS	NS	0.020
Vigorous PA score				
T1 ‘low’	1	1	1	1
T2 ‘middle’	1.21 (0.89–1.65)	1.20 (0.88–1.63)	1.17 (0.97–1.40)	1.13 (0.94–1.35)
T3 ‘high’	**2.11** (1.57–2.83) ^b^	**2.02** (1.50–2.71) ^b^	**1.21** (1.01–1.45)	1.17 (0.98–1.41)
*P for trend*	<0.0001	0.0001	NS	NS
Age group				
30–39	1	1	1	1
40–49	0.75 (0.56–1.00)	**0.73** (0.54–0.99)	**0.45** (0.37–0.55)	**0.47** (0.38–0.57)
50–59	**0.36** (0.25–0.53) ^b^	**0.36** (0.24–0.53) ^b^	**0.22** (0.17–0.28) ^b^	**0.22** (0.17–0.28) ^b^
60–69	**0.33** (0.21–0.53) ^b^	**0.33** (0.21–0.54) ^b^	**0.10** (0.07–0.13) ^b^	**0.09** (0.07–0.13) ^b^
*P for trend*	<0.0001	<0.0001	<0.0001	<0.0001
BMI group				
30–34.9	1	1	1	
≥ 35	**0.37** (0.24–0.55) ^b^	**0.33** (0.22–0.50) ^b^	**0.38** (0.32–0.45) ^b^	**0.37** (0.31–0.44) ^b^
*P for trend*	<0.0001	<0.0001	<0.0001	<0.0001
Smoking				
Non		1		1
Former		0.77 (0.58–1.00)		0.97 (0.82–1.14)
Current		**0.45** (0.32–0.63) ^b^		**0.52** (0.42–0.65) ^b^
Education level				
Low		1		1
Middle		0.99 (0.75–1.30)		0.96 (0.82–1.16)
High		0.88 (0.63–1.24)		**1.30** (1.04–1.63)
Use of a (self-) prescribed diet				
No		1		1
Yes		1.42 (0.86–2.36)		**0.68** (0.53–0.87) ^a^
Energy intake*		0.84 (0.53–1.33)		0.95 (0.72–1.36)

Data are expressed as odds ratios (95% confidence interval). Reference group is the metabolically unhealthy obese. Values in BOLD indicate a P ≤ 0.05, ^a^ denotes a P ≤ 0.01 and ^b^ denotes a P ≤ 0.0001. P for trend test was performed by using for the dietary patterns the median value of each quartile, and a continuous score for physical activity, age and BMI. * Energy intake is given per 1.000 kcal increase. Model 1 (dietary patterns + physical activity): adjusted for age group and BMI group. Model 2 (dietary patterns + physical activity): adjusted for age group, BMI group, smoking, education level, use of a (self)prescribed diet and energy intake

We used the WHO cut-off of ≥6.1 mmol/L for impaired fasting glucose [23]. However, in a subsequent analysis we applied the lower cut-off of ≥5.6 mmol/L for impaired fasting glucose to define metabolic health [32]. This resulted in 196 fewer individuals classified as MHO and 566 more individuals classified as MUO. The observed OR were slightly higher (generally first decimal place increased), for both men and women, using the lower threshold. No new associations were found (data not shown).

## Discussion

In this study more than half of the obese men and more than one third of obese women were metabolically unhealthy. Only 10% of men, and 25% of women were metabolically healthy obese. Compared to those with MUO, we found that women with MHO had a healthier diet, rich in fruit, vegetables, fish and unsweetened fermented milk products while avoiding high sugar beverages and, savory- and sweet snackfood. Women with MHO engaged in more intensive moderate physical activity, while men with MHO were characterized by higher engagement in intensive vigorous physical activity.

Consistent with previous data, total intake of energy and macronutrients did not differ between subjects with MHO and subjects with MUO [12–15]. We identified four major obesity-specific dietary patterns of which only two were associated with MHO, and in women only. The *‘fruit, vegetables and fish’* pattern was positively associated with MHO, and mainly consisted of foods considered as healthy. Previous studies showed that higher intakes of vegetables and fruit were associated with a lower risk of MetS, CVD [33], and only the latter with lower risk of T2D [34, 35]. Higher scores on the *‘fruit, vegetables and fish’* pattern mean higher intake of fish, chicken, unsweetened fermented milk products and low-fat cheese. Both epidemiological and experimental data show that the consumption of dairy products have a beneficial effect on MetS risk factors and are associated with a lower risk of body fat gain and obesity, as well as CVD [36]. Consumption of cheese and fermented dairy product were inversely associated with T2D incidence [37]. The pattern of *‘bread, potatoes and sweet snacks’* was inversely associated with MHO in our study. The pattern consists of carbohydrate-rich food products, mainly refined, which are known to have a high glycaemic index and a low fiber content [38]. Indeed, previous studies have reported an inverse association between high glycaemic index diets and HDL-C [39, 40] and a positive association with triglycerides [41] and MetS [42]. Furthermore, higher glycaemic index and low fiber content are relevant aspects related to insulin resistance and impaired glucose tolerance, which are important co-morbidities of MetS [43].

To date, only a few studies examined dietary patterns and metabolic health in obesity specifically. In two studies ‘a priori’ dietary scores were applied [15, 17]. A study in the US found a higher total score on the HEI-2005 (Healthy Eating Index) in MHO adolescents and adult women compared with metabolically abnormal obese, e.g. MUO [17]. In an Irish cohort study, there was no association between the DASH score (Dietary Approaches to Stop Hypertension)) or the food pyramid score and MHO in adjusted analysis (for sex, age, physical activity, alcohol, smoking and dietary quality) [15]. To the best of our knowledge there is only one study by Bell et al. [44], which used the ‘a posteriori’ approach like in this study. The authors found that the odds of having a more metabolically healthy profile was 16% greater for every standard deviation increase in the ‘healthy’ dietary pattern score (including high loadings of whole grains, fresh fruit, dried fruit, legumes and low fat dairy) [44]. The criteria for metabolic health between our study and the one by Bell et al. [44] were different and dietary patterns were in the latter based on a population including non-obese and obese participants. Still, the outcomes of both studies make us hypothesize that without a calorie restricted diet, ‘healthy’ changes in the dietary pattern may improve metabolic health risk.

Our findings for dietary patterns and MHO were observed for women only. Such a sex difference may reflect differences in physiology, reporting of diet, or the amount consumed of the specific types of foods that contributed strongly to the pattern score [45]. Indeed, higher pattern scores in women also correlated with higher intakes (absolute and/or relative) of the specific foods likely to be associated with metabolic (un)health (Additional file 6).

Increasing one’s physical activity has the potential to improve adiposity profile and metabolic risk, even in the absence of weight loss [46]. A more favourable fat distribution, with less visceral fat, was associated with a long-term metabolically healthy profile in obese adults over a period of 10 year, and no excess risk of T2D and CVD [47]. Other studies on physical activity and MHO found that both objectively measured physical activity [48] and self-reported moderate-vigorous physical activity were higher in the MHO group compared to MUO group [49–51]. In the present study, moderate physical activity in women and vigorous physical activity in men was an important feature in the relationship between physical activity and MHO. In general, men engaged in more physical activity compared to women. To illustrate this, within the ‘fruit, vegetables and fish’ pattern and the ‘bread and potatoes’ pattern men engaged in more moderate to vigorous physical activity compared to women. At the same time men were also more physical active in the lowest vs. highest quartile of the ‘fruit, vegetables and fish’ pattern, and more physical active in the highest vs. lowest quartile of the ‘bread and potatoes’ pattern. This was not seen in women (data not shown). We hypothesize that this results in the stronger positive association between vigorous physical activity and MHO seen in men. Studies using accelerometers will be useful to differentiate physical activity patterns in MHO and MUO separately for men and women.

Our study includes a representative sample of the Dutch population using extensive questionnaires to measure important lifestyle behaviors, and standardized protocols to obtain clinical and biochemical measurements. Another strength of our study is the use of obesity-specific dietary patterns and that it is the largest study on this topic to date.

A limitation of the study includes the use of self-reported data, which are subject to recall bias. Obese individuals tend to underestimate their dietary intake and overestimate their physical activity [52, 53]. However, in our study we only used obese subjects to make comparisons, hence, we ranked individuals into categories which reduces the effect of over- or under-reporting.

PCA is extensively used in nutritional epidemiology and showed reasonable reproducibility and validity using FFQ data [54–57]. It remains, however, a data driven approach. Derived patterns may therefore not be reproducible across studies. Not only the dietary variables can differ across studies, also the process to select the final dietary patterns requires several arbitrary decisions [27]. Yet, it was also found that derived dietary patterns are robust for the subjective factor analytical decisions [58].

Next, we utilized cross-sectional data from which we cannot infer causality. It is, therefore, not possible to rule out reverse causality. Age played an important role within and between the dietary patterns. Furthermore, individuals which experience negative health outcomes, such as having T2D, a CVD history or the knowledge of having dyslipidaemia or hypertension, may have changed their dietary intake and their physical activity. We consider it likely that dietary patterns are not isolated, but part of a broader lifestyle and subject to someone’s phase in life. Furthermore, it was not possible to tease apart the influence of obesity duration on the described associations.

In the literature MHO is still defined by the different classification systems that use different methods to measure metabolic abnormalities as well as different cut-off points or set of parameters to define MetS and/or its components. This makes it difficult to compare and interpret findings of studies on MHO. We defined previously a harmonized set of criteria for MHO within an international collaborative project (BioSHaRE-EU Healthy Obesity Project) [11]. The definition is based on the most widely used criteria for MetS (NCEP ATPIII) and also strict regarding metabolic health status (i.e. according to the classification none of the metabolic disturbances may be present (except for waist circumference) and no history of CVD). A major strength of our definition for MHO is that it can be easily applied in clinical practice and it may also have a greater utility to estimate differences in risk for T2D, CVD and mortality. Future work with longitudinal data should be encouraged to examine the influence of (changes in) dietary patterns on developing adverse metabolic outcomes and more ideally to examine the effect on hard endpoints (i.e. T2D, CVD and mortality).

## Conclusion

In conclusion, our research showed that key lifestyle behaviors differed between metabolically healthy obese and metabolically unhealthy obese adults aged 30–69 years. Non-modifiable factors like age and sex are important for determining someone’s baseline odds for MHO. However, our data suggests that in women, the healthier diet -characterized by *‘fruit, vegetables and fish’*- and moderate physical activity, and in men, vigorous physical activity may be related to this favourable obesity state. The (refined) carbohydrate-rich *‘bread, potatoes and sweet snacks’* dietary pattern was found to counteract MHO in women. Identification of behavioural lifestyle patterns may help in pinpointing vulnerable subgroups in the obese population and to develop potential strategies improving metabolic health.

## Additional files

Additional file 1: Definition for metabolically healthy obesity (MHO), intermediate obesity and metabolically unhealthy obesity. (MUO) (DOCX 20 kb)

Additional file 2: Detailed information on the food items grouping. (DOCX 23 kb)

Additional file 3: Scree plot resulting from principal component analysis. (DOCX 35 kb)

Additional file 4: Loadings of the food groups on the dietary patterns. (DOCX 27 kb)

Additional file 5: Dietary pattern scores across age groups (left panel men, right panel women). (DOCX 1425 kb)

Additional file 6: Mean intake of food groups highly correlated with the dietary pattern, in men and women. (DOCX 24 kb)

## Abbreviations

BMI: body mass index
BP: blood pressure
CVD: cardiovascular disease
EI: energy intake
FFQ: food frequency questionnaire
HDL: high-density lipoprotein
MET: metabolic equivalent
MetS: metabolic syndrome
MHO: metabolically healthy obesity
MUO: metabolically unhealthy obesity
MVPA: moderate-vigorous physical active
PA: physical activity
PCA: principal component analysis
