# Supplementary material for: Dietary patterns and physical activity in the metabolically (un)healthy obese: the Dutch Lifelines cohort study
Source: Nutr J. 2018 Feb 12;17:18. doi: 10.1186/s12937-018-0319-0 (PMC5809859; doi:10.1186/s12937-018-0319-0)
Supplement: Supplementary file 1 — Definition for metabolically healthy obesity (MHO), intermediate obesity and metabolically unhealthy obesity. (MUO) (DOCX 20 kb) [file 12937_2018_319_MOESM1_ESM.docx]

Dietary patterns and physical activity in the metabolically (un)healthy obese: The Dutch Lifelines Cohort Study

Sandra N. Slagter ^1*^, Eva Corpeleijn ^2^, Melanie M. van der Klauw ^1^, Anna Sijtsma ^3^, Linda G. Swart-Busscher ^4^, Corine W.M. Perenboom ^5^, Jeanne H.M. de Vries^5^, Edith J.M. Feskens ^5^, Bruce H.R. Wolffenbuttel ^1^, Daan Kromhout ^2^, Jana V. van Vliet-Ostaptchouk ^1^

*^1^ Department of Endocrinology, University of Groningen, University Medical Center Groningen, PO Box 30001, 9700 RB Groningen, The Netherlands.*

*^2^ Department of Epidemiology, University of Groningen, University Medical Center Groningen, PO Box 30001, 9700 RB Groningen, The Netherlands.*

*^3^ Lifelines Cohort Study, University of Groningen, University Medical Center Groningen, PO Box 30001, 9700 RB Groningen, The Netherlands.*

*^4^ Department of Paramedical Sciences, University of Groningen, University Medical Center Groningen, PO Box 30001, 9700 RB Groningen, The Netherlands.*

*^5^ Division of Human Nutrition, Wageningen University, PO Box 17, 6700 AA Wageningen, The Netherlands.*

*Corresponding author
Sandra N. Slagter, PhD
Dept. of Endocrinology
University of Groningen, University Medical Center Groningen
HPC AA31
P.O. Box 30001
9700 RB Groningen
The Netherlands
Phone: +31 - 50 – 3611483
Fax: +31 - 50 – 3619392
E-mail: [s.n.slagter@umcg.nl](mailto:s.n.slagter@umcg.nl)

Additional file 1. Definition for metabolically healthy obesity (MHO), intermediate obesity and metabolically unhealthy obesity (MUO)

|  | MHO | Intermediate | | MUO |
| --- | --- | --- | --- | --- |
| BMI | ≥30 kg/m^2^ | ≥30 kg/m^2^ | | ≥30 kg/m^2^ |
| MetS risk factor | none | 1 risk factor | | ≥2 risk factors |
| Diagnosis for CVD | no |  | |  |
| **MetS risk factor** |  | | Threshold | |
| Elevated blood pressure |  | | SBP ≥ 130 mmHg or DBP ≥ 85 mmHg or use of antihypertensive medication | |
| Impaired fasting glucose |  | | fasting blood glucose ≥ 6.1 mmol/L or use of blood glucose lowering medication or diagnosis of type 2 diabetes ^a^ | |
| Decreased HDL-cholesterol ^b^ |  | | < 1.03 mmol/L in men or < 1.30 mmol/L in women or medical treatment for low HDL-C | |
| Elevated triglycerides ^b^ |  | | ≥ 1.70 mmol/L or medication for elevated triglycerides | |

Abbreviations: BMI= body mass index, MetS= metabolic syndrome, CVD= cardiovascular disease, DBP= diastolic blood pressure, HDL= high density lipoprotein cholesterol, SBP= systolic blood pressure. ^a^ Diagnosis of type 2 diabetes was based on self-report and verified with self-reported medication use. ^b^ Subjects taking fibrates and/or nicotinic acid are presumed to have either high triglycerides and/or low HDL cholesterol.
