# Supplementary material for: Dietary patterns and physical activity in the metabolically (un)healthy obese: the Dutch Lifelines cohort study
Source: Nutr J. 2018 Feb 12;17:18. doi: 10.1186/s12937-018-0319-0 (PMC5809859; doi:10.1186/s12937-018-0319-0)
Supplement: Supplementary file 2 — Detailed information on the food items grouping. (DOCX 23 kb) [file 12937_2018_319_MOESM2_ESM.docx]

Dietary patterns and physical activity in the metabolically (un)healthy obese: The Dutch Lifelines Cohort Study

Sandra N. Slagter ^1*^, Eva Corpeleijn ^2^, Melanie M. van der Klauw ^1^, Anna Sijtsma ^3^, Linda G. Swart-Busscher ^4^, Corine W.M. Perenboom ^5^, Jeanne H.M. de Vries^5^, Edith J.M. Feskens ^5^, Bruce H.R. Wolffenbuttel ^1^, Daan Kromhout ^2^, Jana V. van Vliet-Ostaptchouk ^1^

*^1^ Department of Endocrinology, University of Groningen, University Medical Center Groningen, PO Box 30001, 9700 RB Groningen, The Netherlands.*

*^2^ Department of Epidemiology, University of Groningen, University Medical Center Groningen, PO Box 30001, 9700 RB Groningen, The Netherlands.*

*^3^ Lifelines Cohort Study, University of Groningen, University Medical Center Groningen, PO Box 30001, 9700 RB Groningen, The Netherlands.*

*^4^ Department of Paramedical Sciences, University of Groningen, University Medical Center Groningen, PO Box 30001, 9700 RB Groningen, The Netherlands.*

*^5^ Division of Human Nutrition, Wageningen University, PO Box 17, 6700 AA Wageningen, The Netherlands.*

*Corresponding author
Sandra N. Slagter, PhD
Dept. of Endocrinology
University of Groningen, University Medical Center Groningen
HPC AA31
P.O. Box 30001
9700 RB Groningen
The Netherlands
Phone: +31 - 50 – 3611483
Fax: +31 - 50 – 3619392
E-mail: [s.n.slagter@umcg.nl](mailto:s.n.slagter@umcg.nl)

Additional file 2. Detailed information on the food items grouping

| Food groups | Food items |
| --- | --- |
| Bread | Bread, crispbread, rusk, croissants and others |
| Rice | Rice |
| Pasta | Pasta |
| Potatoes | Boiled or mashed potatoes |
| Fried potatoes | Fried potatoes |
| Cereals | Muesli, granola or cereals for the preparation of porridges |
| Breakfast drink | Breakfast drink |
| Nonfermented medium/ low-fat milk | Nonfermented medium/ low-fat milk |
| Nonfermented whole milk | Nonfermented whole milk and coffee milk |
|  |  |
| Chocolate milk | Chocolate milk |
| Fermented milk products - unsweetened | Butter milk, full-fat plain yogurt, semi-skimmed plain yogurt, skimmed plain yogurt |
| Fermented milk products - sweetened | Yogurt drinks and flavored dairy drinks with sugar, semi-skimmed fruit or vanilla yogurt, skimmed (fruit) yogurt with sugar |
| Cheese – low fat | 20+ or 30+ cheese or spreadable cheese |
| Cheese – high fat | 40+ or 48+ cheese or spreadable cheese, cream cheese and/or foreign cheese |
| Quark | quark or fruit quark |
| Desserts | (Full-fat) custard and other milk-based desserts |
| Ice cream | Milk-based ice cream |
| Whipped cream | Whipped cream |
| Eggs | Fried and boiled eggs |
| Processed meat | Luncheon meats, hamburger, minced meat (beef or mix of beef and pork), smoked sausages or frankfurters |
| Lean red meat | Beef steak, steak tartare, braising steak or roast beef |
| Red meat | Sirloin steak, beef bratwurst, beef blade steak, beef rib steak or steaked/marbled beef, bacon, pork bratwurst, ‘slavink’ (ground meat wrapped in bacon), pork chops (shoulder, rib, and loin chops) |
| Chicken | Chicken with and without skin |
| Fatty fish | Salted herring, fried herring, salmon, mackerel, eel etc. |
| Lean fish | Cod, plaice, haddock, pollack, sole, deep-fried whiting in dough etc. |
|  |  |
| Commercially prepared dishes | Chinese/Indonesian dishes, meals from fast-food restaurants, other types of ready-to-eat meals |
| Pizza | Ready-to-eat pizza's, homemade pizza's and pizza's eaten at restaurants |
| Warm savory snacks | Croquettes, minced meat hot dogs, sausage rolls |
| Savory snacks | Potato chips or salty biscuits |
| Composed foods | Salads on bread |
| Edible fat | Butter, margarine, low fat margarine |
| Gravy | Gravy |
| Warm sauces | Pasta sauce, mushroom sauce, sate sauce |

| Food groups | Food items |
| --- | --- |
| Mayonnaise | Mayonnaise |
| Non-red sauces | Low fat mayonnaise, sauce for French fries and other non-red sauces |
| Salad dressing | Salad dressing with/without oil |
| Vegetables | Vegetables |
| Fruit | Fesh fruit |
| Apple sauce | Apple sauce |
| Legumes | Brown beans, white beans, marrowfat peas, kidney beans etc. |
| Soup | Soup |
| Peanuts, nuts and seeds | Peanuts, coated peanuts, nuts, seeds and peanut butter |
| Biscuits | Small cookies or (nutritional) biscuits |
| Pastries | Sponge cake, large cookies, cake, pie |
| Candybar | Candybars (Mars, Snickers, M&M's etc.) |
| Chocolate | Chocolate, candy with chocolate and chocolates |
| Candy | Liquorice, acid drops etc. |
| Sweet sandwich toppings | Sweet sandwich toppings (chocolate- sprinkles, spread or flakes, honey, jam) |
| Added sugar | Sugar, honey or syrups |
| Coffee | Coffee |
| Tea | Tea |
| High sugar beverages | Soft drinks (coke, orange flavored soft drinks, 7-up) or lemonade with sugar |
| Low sugar beverages | Diet soft drinks or lemonade without sugar |
| Fruit juices | Fruit juices |
| Beer - light | Non-alcoholic beer |
| Beer | Beer |
| Wine and fortified wine | White-, rosé- and red wine, sherry, port wine or vermouth |
| Spirits | Distilled drinks (genever, whisky, rum, gin, cognac, vieux, liqueur) |
