# Supplementary material for: Dietary patterns and physical activity in the metabolically (un)healthy obese: the Dutch Lifelines cohort study
Source: Nutr J. 2018 Feb 12;17:18. doi: 10.1186/s12937-018-0319-0 (PMC5809859; doi:10.1186/s12937-018-0319-0)
Supplement: Supplementary file 3 — Scree plot resulting from principal component analysis. (DOCX 35 kb) [file 12937_2018_319_MOESM3_ESM.docx]

Dietary patterns and physical activity in the metabolically (un)healthy obese: The Dutch Lifelines Cohort Study

Sandra N. Slagter ^1*^, Eva Corpeleijn ^2^, Melanie M. van der Klauw ^1^, Anna Sijtsma ^3^, Linda G. Swart-Busscher ^4^, Corine W.M. Perenboom ^5^, Jeanne H.M. de Vries^5^, Edith J.M. Feskens ^5^, Bruce H.R. Wolffenbuttel ^1^, Daan Kromhout ^2^, Jana V. van Vliet-Ostaptchouk ^1^

*^1^ Department of Endocrinology, University of Groningen, University Medical Center Groningen, PO Box 30001, 9700 RB Groningen, The Netherlands.*

*^2^ Department of Epidemiology, University of Groningen, University Medical Center Groningen, PO Box 30001, 9700 RB Groningen, The Netherlands.*

*^3^ Lifelines Cohort Study, University of Groningen, University Medical Center Groningen, PO Box 30001, 9700 RB Groningen, The Netherlands.*

*^4^ Department of Paramedical Sciences, University of Groningen, University Medical Center Groningen, PO Box 30001, 9700 RB Groningen, The Netherlands.*

*^5^ Division of Human Nutrition, Wageningen University, PO Box 17, 6700 AA Wageningen, The Netherlands.*

*Corresponding author
Sandra N. Slagter, PhD
Dept. of Endocrinology
University of Groningen, University Medical Center Groningen
HPC AA31
P.O. Box 30001
9700 RB Groningen
The Netherlands
Phone: +31 - 50 – 3611483
Fax: +31 - 50 – 3619392
E-mail: [s.n.slagter@umcg.nl](mailto:s.n.slagter@umcg.nl)

**
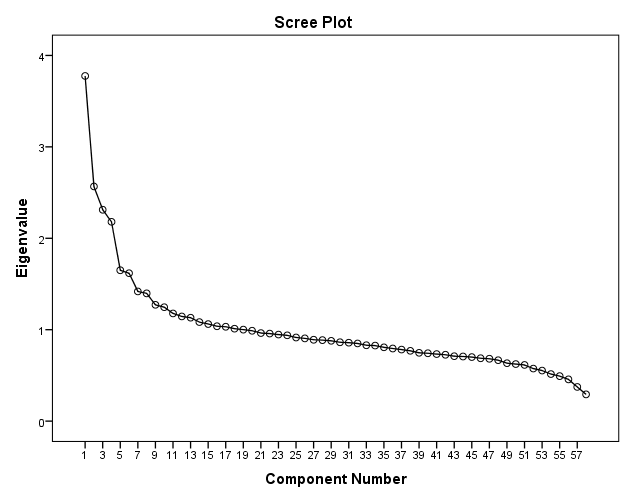
Additional file 3.** Scree plot resulting from principal component analysis
