# Supplementary material for: Dietary patterns and physical activity in the metabolically (un)healthy obese: the Dutch Lifelines cohort study
Source: Nutr J. 2018 Feb 12;17:18. doi: 10.1186/s12937-018-0319-0 (PMC5809859; doi:10.1186/s12937-018-0319-0)
Supplement: Supplementary file 4 — Loadings of the food groups on the dietary patterns. (DOCX 27 kb) [file 12937_2018_319_MOESM4_ESM.docx]

Dietary patterns and physical activity in the metabolically (un)healthy obese: The Dutch Lifelines Cohort Study

Sandra N. Slagter ^1*^, Eva Corpeleijn ^2^, Melanie M. van der Klauw ^1^, Anna Sijtsma ^3^, Linda G. Swart-Busscher ^4^, Corine W.M. Perenboom ^5^, Jeanne H.M. de Vries^5^, Edith J.M. Feskens ^5^, Bruce H.R. Wolffenbuttel ^1^, Daan Kromhout ^2^, Jana V. van Vliet-Ostaptchouk ^1^

*^1^ Department of Endocrinology, University of Groningen, University Medical Center Groningen, PO Box 30001, 9700 RB Groningen, The Netherlands.*

*^2^ Department of Epidemiology, University of Groningen, University Medical Center Groningen, PO Box 30001, 9700 RB Groningen, The Netherlands.*

*^3^ Lifelines Cohort Study, University of Groningen, University Medical Center Groningen, PO Box 30001, 9700 RB Groningen, The Netherlands.*

*^4^ Department of Paramedical Sciences, University of Groningen, University Medical Center Groningen, PO Box 30001, 9700 RB Groningen, The Netherlands.*

*^5^ Division of Human Nutrition, Wageningen University, PO Box 17, 6700 AA Wageningen, The Netherlands.*

*Corresponding author
Sandra N. Slagter, PhD
Dept. of Endocrinology
University of Groningen, University Medical Center Groningen
HPC AA31
P.O. Box 30001
9700 RB Groningen
The Netherlands
Phone: +31 - 50 – 3611483
Fax: +31 - 50 – 3619392
E-mail: [s.n.slagter@umcg.nl](mailto:s.n.slagter@umcg.nl)

Additional file 4. Loadings of the food groups on the dietary patterns

| **Food group** | **Savory snacks and sweets** | **Meat and**  **alcohol** | **Bread, potatoes and sweet snacks** | **Fruit, vegetables and fish** |
| --- | --- | --- | --- | --- |
| Warm sauces | **,551** | ,132 | -,021 | ,136 |
| Savory snacks | **,500** | -,007 | ,030 | -,107 |
| Fried potatoes | **,470** | ,128 | ,058 | -,186 |
| Pasta | **,459** | ,151 | ,027 | ,188 |
| Chocolate | **,431** | -,210 | ,093 | ,092 |
| Non-red sauces | **,415** | ,069 | ,058 | -,192 |
| Commercially prepared dishes (ready to eat meals) | **,398** | ,032 | -,189 | -,111 |
| Pastries | **,384** | -,087 | **,313** | -,037 |
| Candybar | **,383** | -,134 | ,082 | -,075 |
| Pizza | **,377** | ,022 | -,124 | -,127 |
| Composed foods | **,347** | ,156 | -,022 | -,019 |
| Rice | **,343** | ,142 | -,051 | ,297 |
| Candy | **,342** | -,089 | ,111 | -,023 |
| Salad dressing | **,313** | ,116 | -,117 | ,161 |
| Peanuts, nuts and seeds | ,292 | ,163 | -,006 | ,057 |
| Wipped cream | ,267 | ,027 | ,120 | ,012 |
| Ice cream | ,247 | -,022 | ,040 | ,011 |
| Low sugar bevareges | ,219 | -,008 | -,016 | -,065 |
| Fruit juices | ,134 | ,002 | ,025 | -,093 |
| Processed meat | ,137 | **,538** | ,205 | -,020 |
| Beer | ,017 | **,434** | -,086 | -,287 |
| Red meat | -,004 | **,429** | ,214 | -,047 |
| Coffee | -,072 | **,415** | ,012 | -,109 |
| Spirits | ,055 | **,389** | -,138 | -,118 |
| Wine and fortified wine | -,013 | **,318** | **-,301** | ,165 |
| Eggs | ,043 | **,313** | -,224 | ,135 |
| Lean red meat | ,092 | **,304** | ,018 | ,130 |
| Legumes | ,021 | ,271 | ,061 | ,039 |
| Cheese – high fat | ,152 | ,261 | ,013 | -,014 |
| Soup | ,059 | ,198 | -,040 | ,072 |
| Beer – light | -,002 | ,118 | -,039 | -,048 |
| Bread | ,015 | **,302** | **,599** | -,037 |

| **Food group** | **Savory snacks and sweets** | **Meat and**  **alcohol** | **Bread, potatoes and sweet snacks** | **Fruit, vegetables and fish** |
| --- | --- | --- | --- | --- |
| Edible fat | ,010 | ,299 | **,591** | -,045 |
| Potatoes | -,099 | **,304** | **,570** | ,015 |
| Sweet sandwich toppings | ,094 | -,185 | **,498** | ,077 |
| Gravy | -,049 | **,340** | **,486** | -,076 |
| Biscuits | ,261 | **-,300** | **,328** | ,217 |
| Desserts | -,024 | -,024 | **,301** | ,028 |
| Apple sauce | ,067 | -,085 | ,254 | -,121 |
| Nonfermented medium /low fat milk | ,040 | -,022 | ,172 | ,002 |
| Fermented milk products - sweetened | ,061 | -,093 | ,136 | ,021 |
| Vegetables | -,002 | ,238 | ,118 | **,543** |
| Fruit | -,182 | -,073 | ,091 | **,454** |
| Warm savory snacks | **,417** | ,183 | ,056 | **-,434** |
| High sugar beverages | ,231 | ,046 | ,074 | **-,396** |
| Tea | ,025 | **-,306** | ,093 | **,391** |
| Fatty fish | ,005 | ,187 | -,293 | **,357** |
| Mayonnaise | **,326** | ,177 | -,014 | **-,338** |
| Lean fish | ,057 | ,154 | -,267 | **,336** |
| Fermented milk products - unsweetened | -,085 | -,010 | ,060 | **,322** |
| Added sugar | ,011 | ,113 | ,154 | -,295 |
| Chicken | ,107 | ,169 | -,052 | ,278 |
| Quark | -,003 | -,066 | -,056 | ,239 |
| Cheese – low fat | -,173 | ,030 | ,080 | ,237 |
| Cereals | ,104 | -,104 | -,033 | ,209 |
| Chocolate milk | ,107 | -,096 | ,124 | -,164 |
| Nonfermented whole milk | -,039 | ,092 | ,054 | -,119 |
| Breakfast drink | ,021 | -,011 | -,050 | -,081 |
