# Supplementary material for: Dietary patterns and physical activity in the metabolically (un)healthy obese: the Dutch Lifelines cohort study
Source: Nutr J. 2018 Feb 12;17:18. doi: 10.1186/s12937-018-0319-0 (PMC5809859; doi:10.1186/s12937-018-0319-0)
Supplement: Supplementary file 6 — Mean intake of food groups highly correlated with the dietary pattern, in men and women. (DOCX 24 kb) [file 12937_2018_319_MOESM6_ESM.docx]

Dietary patterns and physical activity in the metabolically (un)healthy obese: The Dutch Lifelines Cohort Study

Sandra N. Slagter ^1*^, Eva Corpeleijn ^2^, Melanie M. van der Klauw ^1^, Anna Sijtsma ^3^, Linda G. Swart-Busscher ^4^, Corine W.M. Perenboom ^5^, Jeanne H.M. de Vries^5^, Edith J.M. Feskens ^5^, Bruce H.R. Wolffenbuttel ^1^, Daan Kromhout ^2^, Jana V. van Vliet-Ostaptchouk ^1^

*^1^ Department of Endocrinology, University of Groningen, University Medical Center Groningen, PO Box 30001, 9700 RB Groningen, The Netherlands.*

*^2^ Department of Epidemiology, University of Groningen, University Medical Center Groningen, PO Box 30001, 9700 RB Groningen, The Netherlands.*

*^3^ Lifelines Cohort Study, University of Groningen, University Medical Center Groningen, PO Box 30001, 9700 RB Groningen, The Netherlands.*

*^4^ Department of Paramedical Sciences, University of Groningen, University Medical Center Groningen, PO Box 30001, 9700 RB Groningen, The Netherlands.*

*^5^ Division of Human Nutrition, Wageningen University, PO Box 17, 6700 AA Wageningen, The Netherlands.*

*Corresponding author
Sandra N. Slagter, PhD
Dept. of Endocrinology
University of Groningen, University Medical Center Groningen
HPC AA31
P.O. Box 30001
9700 RB Groningen
The Netherlands
Phone: +31 - 50 – 3611483
Fax: +31 - 50 – 3619392
E-mail: [s.n.slagter@umcg.nl](mailto:s.n.slagter@umcg.nl)

Additional file 6. Mean intake of food groups highly correlated with the dietary pattern, in men and women

| *Mean intake/day* | **Bread, potatoes and sweet snacks** | | | | **Fruit, vegetables and fish** | | | |
| --- | --- | --- | --- | --- | --- | --- | --- | --- |
|  | *Consumption in gram/day within Q4* | | *Consumption per 1.000 kcal intake within Q4* | | *Consumption in gram/day within Q4* | | *Consumption per 1.000 kcal intake within Q4* | |
|  | **Men** | **Women** | **Men** | **Women** | **Men** | **Women** | **Men** | **Women** |
| Bread | 204.48 | 157.53^a^ | 83.03 | 75.24^a^ |  |  |  |  |
| Sweet sandwich toppings | 17.63 | 20.09^a^ | 7.24 | 9.61^a^ |  |  |  |  |
| Potatoes | 119.18 | 94.39^a^ | 49.46 | 45.85^a^ |  |  |  |  |
| Gravies | 27.39 | 22.52^a^ | 11.35 | 10.91 |  |  |  |  |
| Edible fat | 40.94 | 31.62^a^ | 16.56 | 15.02^a^ |  |  |  |  |
| Biscuits | 18.87 | 24.95^a^ | 7.71 | 11.68^a^ |  |  |  |  |
| Pastries | 24.94 | 26.32 | 9.97 | 12.23^a^ |  |  |  |  |
| Desserts | 64.31 | 57.07^a^ | 26.54 | 27.75 |  |  |  |  |
| Wine and fortified wine | 12.07 | 13.52 | 4.79 | 6.27^a^ |  |  |  |  |
| Fatty fish | 2.50 | 2.30^a^ | 1.00 | 1.07 | 7.41 | 7.06 | 3.96 | 4.59^a^ |
| Lean fish | 6.19 | 6.11^a^ | 2.51 | 2.88^a^ | 12.71 | 12.78 | 6.72 | 8.42^a^ |
| Fermented milk products -unsweeted |  |  |  |  | 91.91 | 108.90^a^ | 47.84 | 67.75^a^ |
| Vegetables |  |  |  |  | 132.73 | 146.83^a^ | 68.45 | 92.84^a^ |
| Fruit |  |  |  |  | 187.98 | 207.84^a^ | 96.52 | 130.89^a^ |
| Tea |  |  |  |  | 222.26 | 399.04^a^ | 116.94 | 255.07^a^ |
| Mayonaise |  |  |  |  | 1.35 | 0.68^a^ | 0.65 | 0.39^a^ |
| High sugar beverages |  |  |  |  | 32.43 | 15.25^a^ | 14.90 | 8.45^a^ |
| Savory snacks |  |  |  |  | 4.46 | 3.52^a^ | 2.06 | 1.93^a^ |
| ^a^ denotes a P <0.01. | | | | | | | | |
|  | | | | | | | | |
